# Why We Must Provide Better Support for Pakistan's Female Frontline Health Workers

**DOI:** 10.1371/journal.pmed.1001528

**Published:** 2013-10-08

**Authors:** Svea Closser, Rashid Jooma

**Affiliations:** 1 Department of Sociology and Anthropology, Middlebury College, Middlebury, Vermont, United States of America; 2 Department of Surgery, Aga Khan University, Karachi, Pakistan

## Abstract

Svea Closser and Rashid Jooma argue that achieving polio eradication and strengthening Pakistan's health system must focus not just on international engagement but also on local partnerships with Lady Health Workers and other ground-level staff.

*Please see later in the article for the Editors' Summary*

**Summary Points**

- As the Global Polio Eradication Initiative deploys its endgame strategy, the commitment and effectiveness of field health workers in polio-endemic countries is critical.
- Ongoing attacks on Lady Health Workers and other frontline health staff in Pakistan appear to be an unintended consequence of the high political profile of polio eradication.
- Achieving polio eradication and strengthening Pakistan's health system now depends on a shift in the center of gravity of international engagement, away from high-profile engagement with federal leaders and towards supportive partnerships with Lady Health Workers and other ground-level staff.
- Nearly all women who work on the health frontline in Pakistan do so because poverty and a lack of other opportunities force them to accept a job with pay of under US$5 per day.
- Steps to support Lady Health Workers and to engage them as strong partners should include paying a living wage, developing world-class security strategies, and providing opportunities for career development and advancement.

## Introduction

Polio is a crippling and deadly viral disease. Through an impressive effort involving millions of people and billions of doses of oral polio vaccine, the Global Polio Eradication Initiative (GPEI) has interrupted polio transmission in all but three countries: Pakistan, Afghanistan, and Nigeria [1].

In Pakistan, weak health systems are a key reason that polio elimination is so difficult [2]–[4]. Rates of routine immunization, which would provide a population-wide firewall against polio, remain low [5].

Critical to the eradication effort in Pakistan are the country's 106,000 Lady Health Workers (LHWs), government health staff who work on vaccination teams in special door-to-door immunization campaigns. But militant groups have begun to target these workers, breaking down the front line of defense against polio.

## Why Are Lady Health Workers Being Killed?

In December 2012, militants murdered 9 polio campaign workers in Pakistan, and the killing has continued into 2013 with the death toll now nearing 20. Workers going door-to-door delivering polio vaccine have been shot, and Lady Health Workers have been the targets of bombings at health centers [6]–[13].

Aid programs generally, and vaccination programs specifically, have become associated with CIA and Western interests in Pakistan. The CIA's tactic of employing a fake vaccination campaign to search for Osama Bin Laden damaged health workers' credibility [14]. The Taliban have targeted LHWs in the Swat Valley, ostensibly for promoting contraceptives and representing Western interests [15].

In this context, polio eradication's high political profile contributed to making the program a militant target. In the past few years, the leadership of the GPEI made a strategic decision to intensely and publicly engage national leaders in the hope of leveraging their perceived authority and oversight for the polio program [16]–[21]. The resulting greater political involvement has increased commitment and accountability [1],[22],[23]. Many observers see this development as wholly positive. However, we believe that the much-publicized importance of polio eradication to national leaders and international organizations helped to make polio eradication workers, including Lady Health Workers, targets of anti-government and anti-state elements [24],[25].

Deep-seated ideological opposition to polio eradication by militants is unlikely: the Taliban, for example, actively support polio vaccination campaigns when it is politically advantageous [26],[27]. Militants might be killing polio workers for political reasons: attacking polio eradication is a way to attack national and international interests.

Here, we argue that achieving polio eradication and strengthening Pakistan's health system depend on a shift in the center of gravity of international engagement, away from high-profile engagement with federal leaders and towards supportive partnerships with Lady Health Workers and other ground-level staff.

## Lady Health Workers Are Needed to Improve Pakistan's Health System

Each Lady Health Worker acts as the interface between around 150 urban slum or rural households and the health system. In addition to polio campaigns, she visits families monthly to promote family planning; advise on nutrition and hygiene; and create demand for antenatal care, childhood immunization, and use of skilled birth attendants. While the program is not perfect, independent evaluations have consistently shown improved health outcomes on a range of primary healthcare measures in populations with LHW coverage. The households served by LHWs are 15 percentage points more likely to have children under 3 years old fully immunized. Over 90% of communities served by LHWs report that they benefit from her services [28],[29].

## Lady Health Workers Are Needed for Polio Eradication

Pakistan's current polio eradication Emergency Action Plan [22] mandates that each of the approximately 80,000 mobile teams deployed in polio campaigns include a female. As there are few other women in the government health system, Lady Health Workers (LHWs) are an essential part of the polio workforce—more than 85% of the total number of LHWs are engaged in each campaign. They contribute to polio eradication through health education and door-to-door delivery of polio vaccine. Most importantly, because most are locally known women, their presence increases access to conservative households and decreases refusals.

Pakistan has decided not to suspend polio campaigns even in the face of danger to LHWs and other workers. This decision has received international approval because halting campaigns would probably mean a resurgence of polio, including possible reinfection of countries like India and China [30]. Decades of painstaking progress might slide away.

In many parts of Pakistan, UN staff who work on polio campaigns have been pulled from the field for their safety. At the same time, LHWs continue to put their lives at risk [31],[32].

Without UN staff in the field, polio eradication needs LHWs more than ever. If eradication is to succeed, particularly in conflict-torn areas, LHWs' full support is essential.

## Lady Health Workers Risk Their Lives, But Do Not Receive a Living Wage

While media accounts of the killings commonly depict female vaccinators as “aid workers” heroically working for the health of others, the reality is more complicated [8],[33]–[35]. LHWs are often in desperate financial straits and work for pay of under US$5 per day because there are few other jobs available to women (Box 1). Financial insecurity is a serious problem for LHWs. It is also a major issue for other women who are hired temporarily a few days a month for polio vaccination campaigns. Married women often become LHWs because their husbands are absent, drug addicts, disabled, or underemployed; unmarried women often take on the work because their father is dead or unemployed and any brothers are unable to financially support the family [36]–[38].

**Box 1. Financial Need and Health Work: A Lady Health Worker's Story**

I became an LHW because of problems at home. I have no choice. I get paid for my blood and my sweat, but there's relief in the work too… Everything is so expensive now, so expensive—but I can scrape by. Thank God.

When I became an LHW, I was responsible for my son. I was living with my parents. My husband had left. He came back after three years, after I'd figured out how to take care of myself, how to take care of my son. And I'm very satisfied that I didn't have to beg from anyone. Sure, my salary was very small, but it was my own money. With that money I took care of myself, I took care of my son, I'm sending him to school. I'm very satisfied with that. My son is seven, *mashallah*, and he's in second grade…

It doesn't matter what your family background is, standing on your own two feet is the most important thing… It's just the first step that someone has to take by themselves. When someone tries, Allah surely will give them rewards for their work, and Allah builds courage in that person.

*[Interview conducted by SC in June 2011.]*

In 2011, SC conducted semi-structured interviews with more than 60 frontline polio workers and supervisors, a sample drawn from all provinces of Pakistan. Detailed information on this study was presented in a report for UNICEF [36]. In these interviews, the vast majority of LHWs and other frontline polio workers said their income was insufficient for basic needs: food, transport, and housing. Most were in families with a rate of income around—or well below—US$2 a day per person. Female polio workers said that low pay dampened their motivation. “When after working so hard you get so little money, your heart breaks,” one woman explained. “And you don't want to do that work. It's the truth” [36].

As many female health workers in Pakistan are the sole or primary source of income for their families, a living wage to ensure food security and availability of school fees for their children is a necessary first step towards engaging them as strong partners. The GPEI, which has an annual budget of over US$1 billion, should prioritize funding for this purpose [39]. At a minimum, workers should receive Rs. 1000 (a little under US$10) per day for the three days a month they work on polio campaigns, and both regular and polio pay should be delivered on time.

A living wage can be the linchpin of a stronger policymaker–LHW partnership, and can have positive effects beyond polio eradication. One analysis of routine immunization coverage in Pakistan recommended changing incentives for health workers and better engaging LHWs [40].

## Building Stronger Partnerships with LHWs Will Lead to True Health System Strengthening and Help Eradicate Polio

### Contribute to Security by Lessening the Visible Involvement of Political Leaders

The induction of a new government in Islamabad after the recent elections presents an opportunity for partners in polio eradication to shift their focus from a “leader-centric” model to one that focuses on gaining the true support and advocacy of grassroots health workers. To contribute to secure conditions for these workers, national engagement must visibly shift away from the federal level. The international stakeholders of the GPEI should, in the near term, restrict high-profile involvement with government functionaries and focus on a new strategy that values, supports, and engages its critical frontline workers.

Such a program could be at once far-reaching and low-profile, building on the existing system for training polio workers to communicate their crucial role in this historic project. This strategy should complement a program of empowerment and career development for these workers.

### Build Capacity by Providing More Opportunities for LHWs

Limited opportunity for career advancement is a real problem for LHWs [36],[41]–[44]. Many LHWs and other polio campaign workers desire more education and the opportunity to advance within the health system, but feel that opportunities to do so are not available (Box 2).

**Box 2. Job Advancement: The Experience of One Lady Health Worker**

I became an LHW when I was very young… I had such desire to become a doctor! There was a dentist here, and I used to go to her office and follow her around and work for her, just out of interest, I wanted to know more about how I could become a doctor… I've been thinking of further study, because really, I want to move up. But I look, and there really aren't any ways for me to advance. There's no chance, absolutely no way. All of my dreams, I've left them all behind. What I wanted to do.

*[Interview conducted by SC in June 2011.]*

Pakistan's Expanded Program on Immunization has recently started training LHWs to administer injectable vaccines in an effort to bolster lagging national immunization rates and augment the efforts of facility-based vaccinators. This promising start could be deepened and expanded by making the LHW responsible for routine vaccinations in her area and making her home, the “Health House” of the community, the vaccination center for her neighborhood [29]. Studies in a variety of settings show that such community-centered vaccination is effective in strengthening routine immunization [45]–[47]. The health establishment of Pakistan should be assisted in gradually increasing the number of LHWs to cover the 40% of rural and urban slum populations presently outside the ambit of the program. Such steps could improve Pakistan's weak routine immunization rates, which would also further the eradication of polio [5].

To make such changes empowering and not exploitative they must be built on improved wages and enhanced support for LHWs from supervisors and district health officers. Problems in the pharmaceutical supply chain and payment provision must be addressed [29],[36],[48]. The recent “regularization” of LHWs, which extends to them the same job security and benefits that other government employees enjoy, along with an increase in regular salary to Rs. 9,000 (about $90) a month, is a major step in the right direction [49]. A program truly engaging LHWs as partners would build on this promising start to craft a program of collaborative engagement that listens to LHWs and takes their needs seriously.

A critical part of supporting LHWs is providing effective security. Some LHWs are refusing the police escorts provided to them, apparently because they make LHWs more visible [12]. This suggests that current security strategies are suboptimal, and that the government machinery alone cannot provide secure conditions for vaccination outreach. Complicating the issue is the fact that the threat to health workers is multidirectional and includes local players in many troubled districts. The GPEI currently employs experts in epidemiology, virology, and communications. Now it is critical that, in addition, the program engage security experts to develop district-wise strategies for providing world-class security to LHWs. These local solutions should be incorporated into the polio campaign plan.

To provide broader support for LHWs, formal opportunities for job advancement should be provided. Pakistan has an acute shortage of nurses [50]–[53]. Opportunities for midwifery, nursing, and other advanced training for Pakistan's most experienced and conscientious LHWs would open doors for hardworking women and add cadres of skilled health staff to Pakistan's health system. Studies have identified opportunities for career advancement and professional development as two important contributors to vibrant community health worker programs [54]–[56]. Tying such opportunities to clearly communicated performance targets would give talented women struggling to support their families fresh reason for commitment.

Pakistan's LHWs have the potential to achieve universal immunization and polio eradication in the country. In fact, both of these goals are probably impossible without their full support. Achieving them depends on a shift from treating frontline female health staff as disposable labor to truly engaging them as well-supported, active partners in achieving a healthier Pakistan.

## Abbreviations

GPEI: Global Polio Eradication Initiative
LHW: Lady Health Worker
